# Dexamethasone Treatment Induces the Reprogramming of Pancreatic Acinar Cells to Hepatocytes and Ductal Cells

**DOI:** 10.1371/journal.pone.0013650

**Published:** 2010-10-27

**Authors:** Amani Al-Adsani, Zoë D. Burke, Daniel Eberhard, Katherine L. Lawrence, Chia-Ning Shen, Anil K. Rustgi, Hiroshi Sakaue, J. Mark Farrant, David Tosh

**Affiliations:** 1 Department of Biology and Biochemistry, Centre for Regenerative Medicine, University of Bath, Bath, United Kingdom; 2 Stem Cell Program, Genomics Research Center, Academia Sinica, Taipei, Taiwan, Republic of China; 3 Department of Medicine and Genetics, Abramson Cancer Center, University of Pennsylvania, Philadelphia, Pennsylvania, United States of America; 4 Department of Nutrition and Metabolism, Institute of Health Biosciences, The University of Tokushima Graduate School, Tokushima, Japan; 5 Department of Gastroenterology, Royal United Hospital, Bath, United Kingdom; The University of Hong Kong, Hong Kong

## Abstract

**Background:**

The pancreatic exocrine cell line AR42J-B13 can be reprogrammed to hepatocytes following treatment with dexamethasone. The question arises whether dexamethasone also has the capacity to induce ductal cells as well as hepatocytes.

**Methodology/Principal Findings:**

AR42J-B13 cells were treated with and without dexamethasone and analyzed for the expression of pancreatic exocrine, hepatocyte and ductal markers. Addition of dexamethasone inhibited pancreatic amylase expression, induced expression of the hepatocyte marker transferrin as well as markers typical of ductal cells: cytokeratin 7 and 19 and the lectin peanut agglutinin. However, the number of ductal cells was low compared to hepatocytes. The proportion of ductal cells was enhanced by culture with dexamethasone and epidermal growth factor (EGF). We established several features of the mechanism underlying the transdifferentiation of pancreatic exocrine cells to ductal cells. Using a CK19 promoter reporter, we show that a proportion of the ductal cells arise from differentiated pancreatic exocrine-like cells. We also examined whether C/EBPβ (a transcription factor important in the conversion of pancreatic cells to hepatocytes) could alter the conversion from acinar cells to a ductal phenotype. Overexpression of an activated form of C/EBPβ in dexamethasone/EGF-treated cells provoked the expression of hepatocyte markers and inhibited the expression of ductal markers. Conversely, ectopic expression of a dominant-negative form of C/EBPβ, liver inhibitory protein, inhibited hepatocyte formation in dexamethasone-treated cultures and enhanced the ductal phenotype.

**Conclusions/Significance:**

These results indicate that hepatocytes and ductal cells may be induced from pancreatic exocrine AR42J-B13 cells following treatment with dexamethasone. The conversion from pancreatic to hepatocyte or ductal cells is dependent upon the expression of C/EBPβ.

## Introduction

Transdifferentiation belongs to the wider class of cell type conversions known as reprogramming [1]. One example of reprogramming is the conversion of pancreatic cells to hepatocytes. The appearance of hepatic foci in adult pancreas has been observed in rodent models and cancer patients [2], [3], [4], [5].

We previously developed an *in vitro* model for studying the reprogramming of pancreatic cells to hepatocytes based on the addition of the synthetic glucocorticoid dexamethasone (Dex) to AR42J-B13 (B13) cells [6], [7]. B13 cells are derived from a rat pancreatic tumour [8] and display both exocrine and neuroendocrine properties [9]. When cultured with Dex for 14 days, pancreatic AR42J-B13 cells begin to express markers typical of hepatocytes [6], [7]. We have recently shown that the phosphoinoside 3-kinase pathway is important in the transdifferentiation of pancreatic acinar cells to hepatocytes and that the hepatocytes arise from acinar cells via an intermediate expressing the ATP-binding cassette sub-family G member 2 (ABCG2) [10]. In addition to hepatocytes, AR42J cells may be induced to also form insulin-producing β-cells following treatment with hepatocyte growth factor (HGF) and activin [11] suggesting the cells may exhibit a progenitor phenotype. The question arises whether other types of cells (apart from hepatocytes), are induced following Dex treatment of AR42J-B13 cells. The reason for specifically examining the ductal phenotype is two-fold. First, during liver development, bipotential hepatoblasts can differentiate towards either hepatocyte or biliary lineages [12]. Second, acinar-ductal transdifferentiation is clinically significant because it may predispose to the development of neoplasia [13]. Transdifferentiation of primary acinar cells to ductal cells occurs when the cells are placed in primary culture [14], [15] Acinar-to-ductal transdifferentiation may occur in experimental pancreatitis and in the progression to pancreatic neoplasia [16], [17], [18], [19].

In models of adult pancreatic regeneration, exocrine acini are found to transdifferentiate to duct-like complexes in a process called acinar-ductal metaplasia (reviewed in [20]). This form of metaplasia is also observed in a model of pancreatic ductal ligation [21]. In the present study we have investigated the potential of AR42J-B13 cells to differentiate towards other cell types (apart from hepatocytes) following Dex treatment. We show that (i) ductal cells are formed in Dex-treated B13 cells, (ii) the number of ductal cells can be increased by treatment with Dex and epidermal growth factor (Dex/EGF) in combination (iii) ductal cells can arise from exocrine (amylase-positive) cells and (iv) overexpression of CCAAT enhancer binding protein β (C/EBPβ), a transcription factor previously shown to mediate hepatocyte transdifferentiation of pancreatic cells, inhibits conversion to a ductal phenotype.

## Results

### Expression of ductal markers in adult rat liver and pancreas

**Figure 1:**
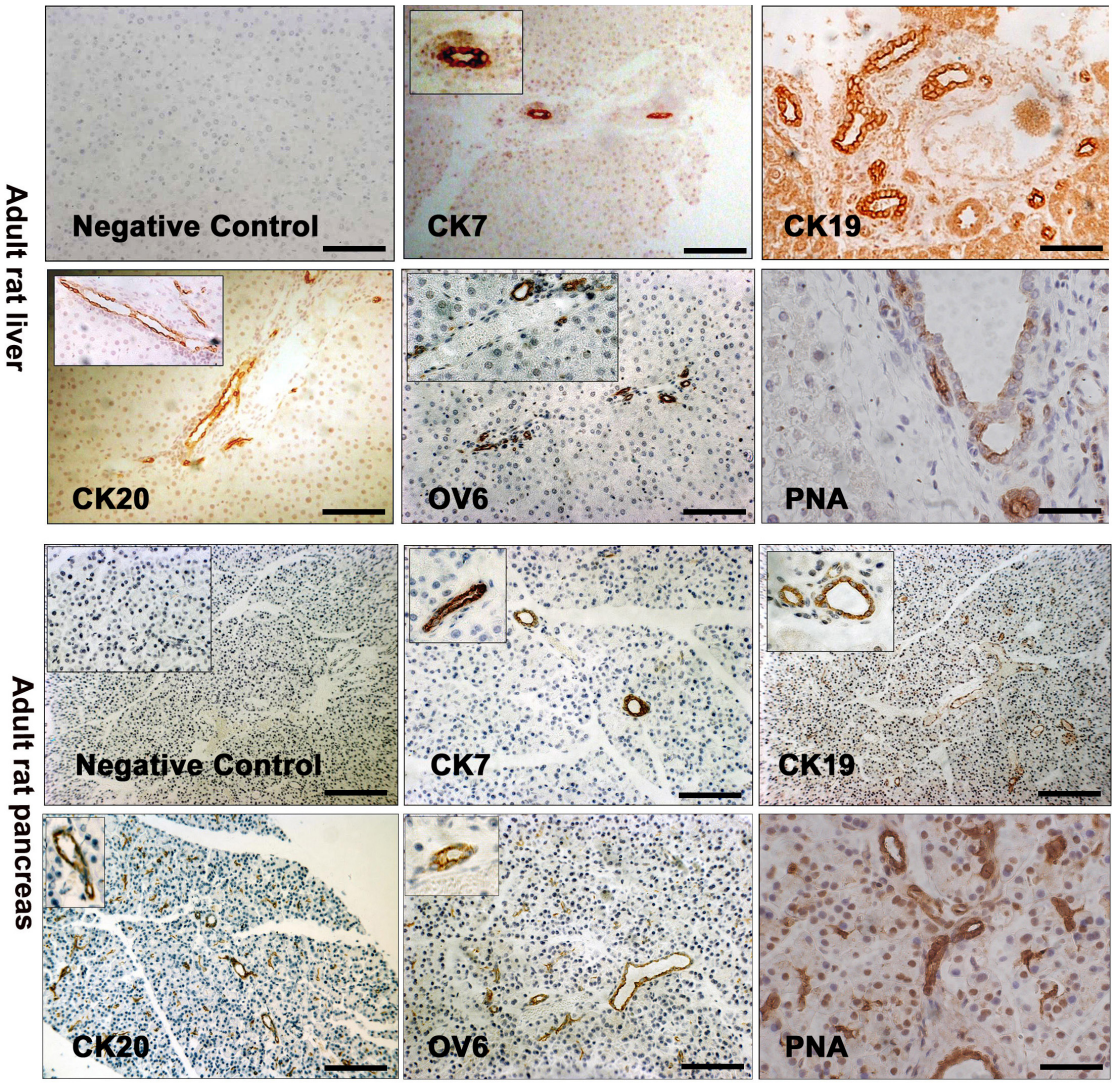
Expression of ductal markers in adult rat liver and pancreas tissue. Immunohistochemistry for cytokeratin 7 (CK7), cytokeratin 19 (CK19), cytokeratin 20 (CK20), OV6 and Peanut Agglutinin (PNA) in adult rat liver and pancreas sections. A control (no primary antibody) is also shown. All scale bars, 100 µm.

In order to test the utility of our antibodies in adult rat liver and pancreas tissue (rat tissue was used because B13 cells are also of rat origin [8]), we determined initially the expression pattern of the markers CK7, CK19, CK20 and OV6 (Figure 1). As expected, the antibodies to the ductal markers CK7, CK19 and CK20 stained the intrahepatic bile ducts (but not hepatocytes) in the liver, whereas the oval cell marker OV6 was only expressed in the smaller bile duct cells as reported previously [20], [21]. In the pancreas, ducts (but not acinar or endocrine cells) were positive for CK7, CK19 and CK20 and OV6 (Figure 1). Ducts in the liver and the pancreas were also positive for the epitope recognised by the lectin Peanut Agglutinin (PNA). Thus PNA can be used as a ductal marker along with cytokeratins 7 and 19 (Figure 1). We did not observe any non-specific staining in the absence of primary antibody (Figure 1). 

### Dex treatment of AR42J-B13 cells induces both hepatocyte and ductal phenotypes

**Figure 2:**
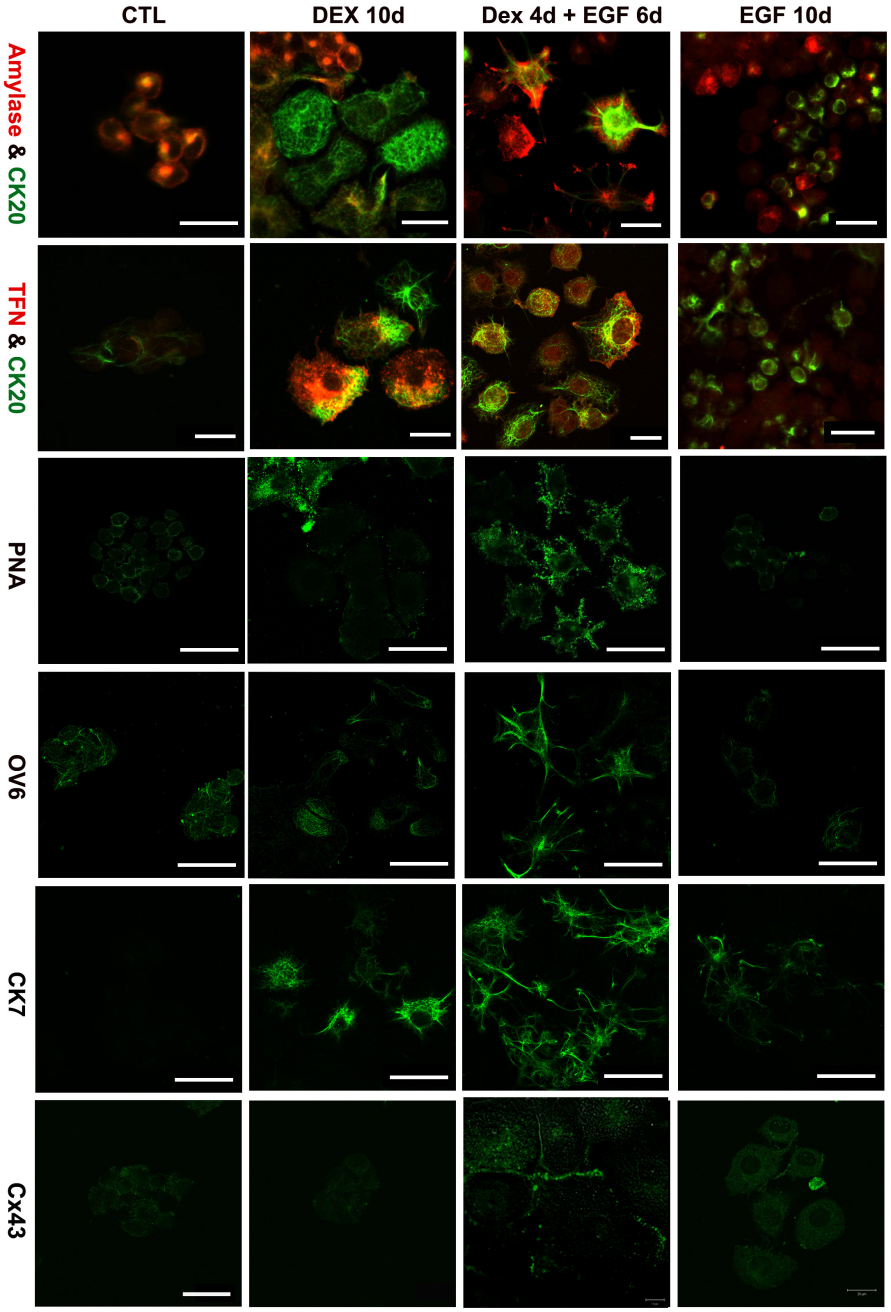
Differentiation of B13 cells to ductal and hepatic phenotypes. Immunostaining for Amylase (red)/CK20 (green), TFN (red)/CK20 (green), PNA, OV6, CK7, Cx43 (green) in untreated (control, CTL), DEX, Dex/EGF and EGF treated B13 cells. Scale bars, first and second row, 20 µm; all others 40 µm.

We cultured B13 cells with or without Dex a period of 10 days and examined the expression of pancreatic exocrine, hepatocyte and ductal markers (Figure 2). The majority of control AR42J-B13 cells co-expressed the exocrine marker amylase and CK20, which labels both progenitor-like and ductal cells [22]. The cells were also weakly positive for OV6 and PNA. Control B13 cells did not express the hepatocyte marker transferrin (TFN) (Figure 2) or the ductal-specific markers CK7 and Connexin 43 (Cx43) (Figure 2).

**Figure 3:**
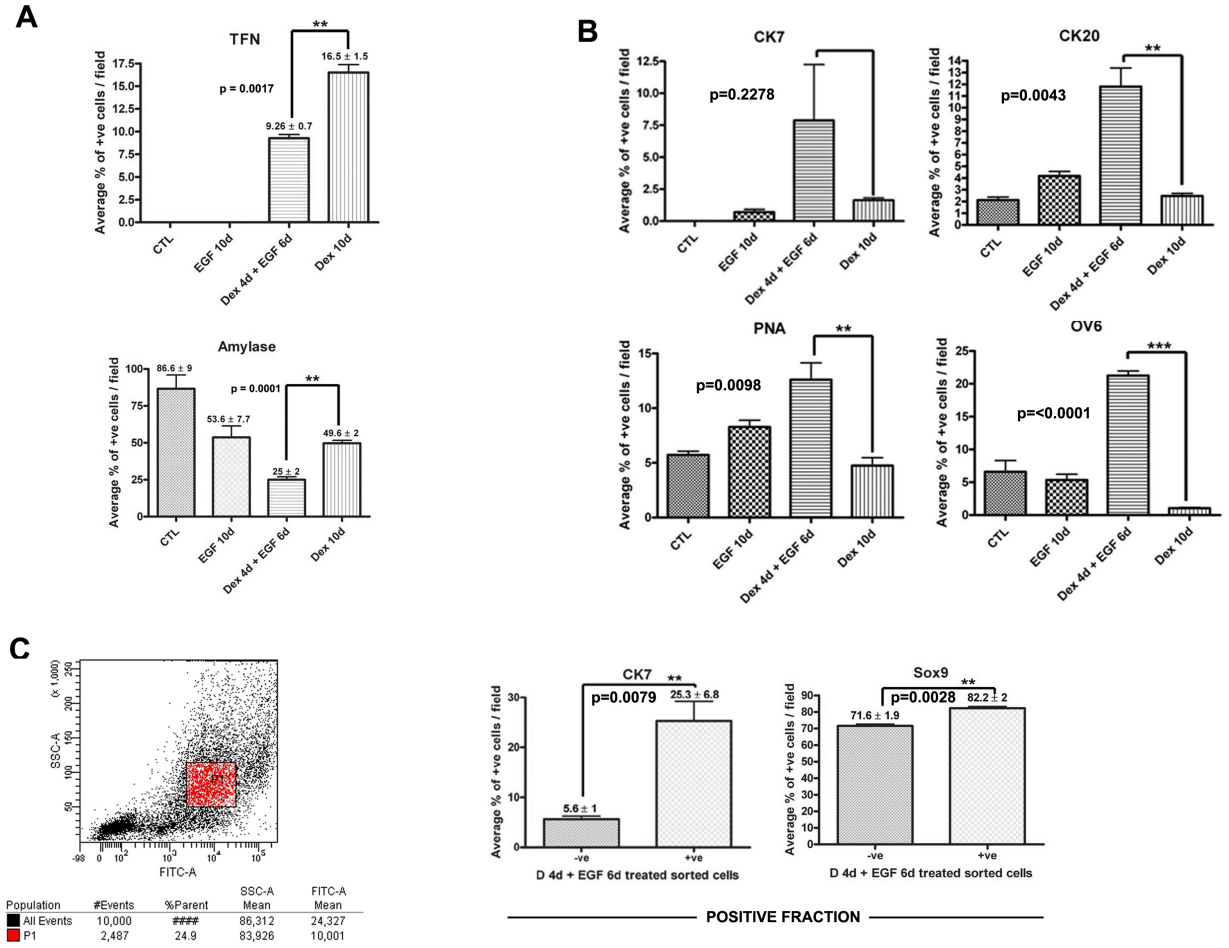
EGF enhances the ductal phenotype at the expense of the hepatic phenotype. Bar charts showing the percentage of cells expressing (A) Amylase and Transferrin (B) CK7, CK20, PNA and OV-6 in control, EGF, Dex/EGF and Dex treated cells. (C) Scatter plot from the FACSCanto showing the intensity of PNA staining in Dex/EGF treated cells and bar charts showing percentage of cells positive for CK7 and Sox9 (positive fraction) following MACS isolation. Scale bars, top and middle row, 20 µm; lower row, 50 µm.

During Dex treatment, amylase expression is lost in AR42J-B13 cells which also begin to express the hepatocyte marker TFN (Figure 3A), as reported previously [6]. After treatment with Dex for 3 days a small number of cells expressing the ductal markers CK7 and CK19 were detected and expression of CK20, OV6 and the binding sites for PNA were enhanced (data not shown). Some cells still expressed CK7, CK20, OV6 and PNA following treatment with Dex for 10 days (Figure 2, Figure 3B). However, only around 2% of the cells were positive for CK7 compared with 16.5% for transferrin in Dex-treated cultures (Figure 3B).

### EGF enhances the number of ductal-like cells in Dex-treated cultures

As the incidence of ductal cells (based on immunostaining for CK7) is uncommon in Dex-treated cultures, we tried to enhance the number of ductal cells by addition of extracellular factors. EGF is one such candidate factor since EGF receptor stimulation in pancreatic tissue induces an acinar to ductal metaplasia [23], [24].

**Figure 4:**
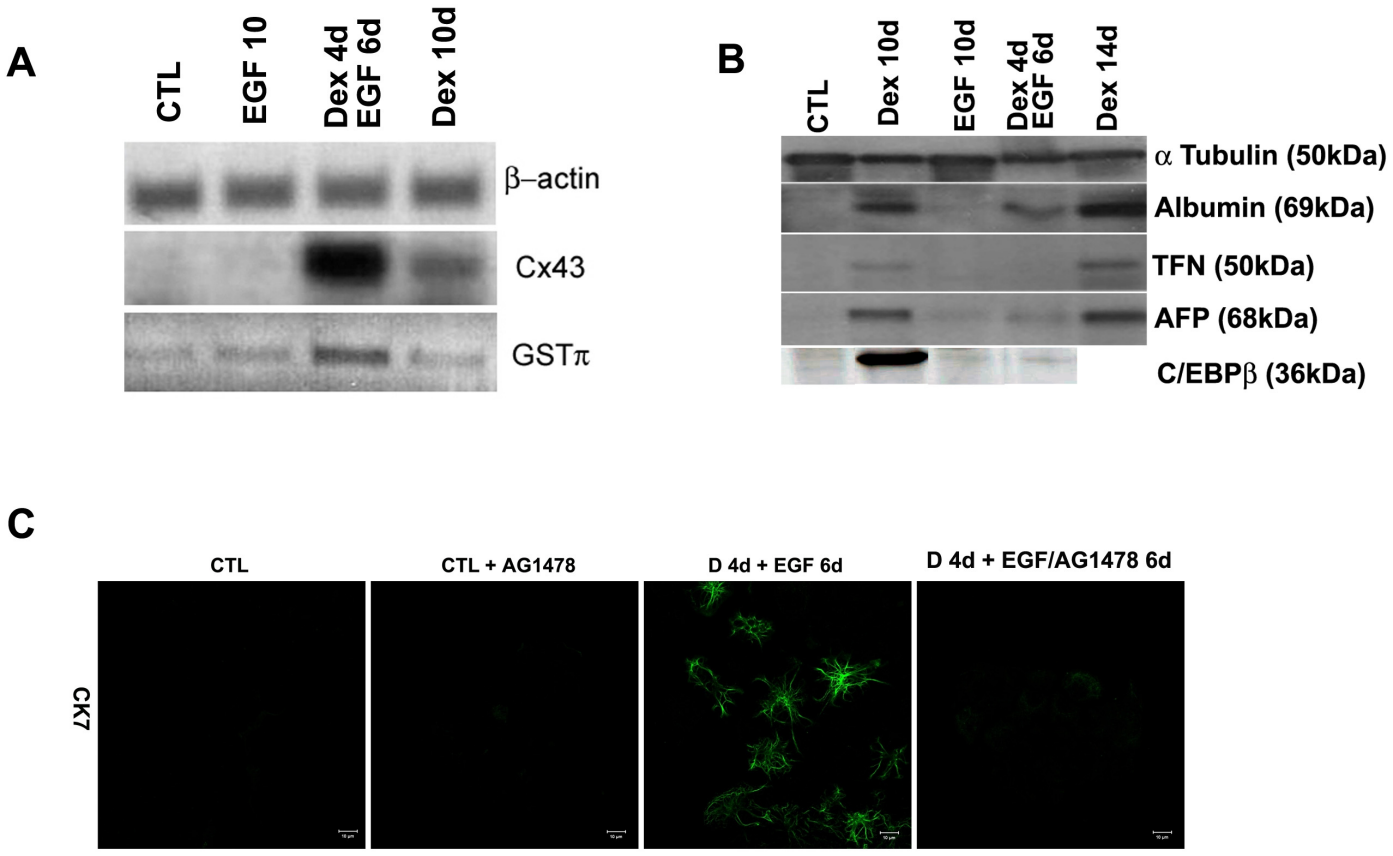
Expression of ductal markers and inhibition of the ductal phenotype. (**A**) RT-PCR for Cx43 and GSTπ (B) Western blotting analysis for Albumin, TFN, AFP and the liver enriched transcription factor C/EBPβ in control, EGF, Dex/EGF and Dex treated cells. β-actin and α-tubulin are also shown as loading controls. (C) Immunostaining for CK7 in control and Dex/EGF treated cells in the presence and absence and absence of the EGF receptor inhibitor AG1478. The inhibitor was added at a final concentration of 25 µM.

We tried different combinations of Dex and EGF treatment on B13 cells. When treated with 1 µM Dex for 4 days followed by 20 ng/ml EGF treatment for 6 days (the combined treatment will be referred to as Dex/EGF from now on), the percentage of cells staining positive for CK7, CK20 and the lectin PNA, increased compared to cells treated with EGF or Dex for 10 days (Figure 2, Figure 3B). In addition the ductal markers Cx43 and GSTπ were detected only in Dex/EGF-treated cells by immunostaining (Cx43) and RT-PCR (Cx43 and GSTπ) (Figure 2 and Figure 4A respectively). Cx43 expression was localised in a punctate pattern on membranes of opposing cells (Figure 2). When B13 cells were treated for 10 days with EGF alone, amylase expression was lost in a proportion of cells (Figure 3A; 53.6% in EGF treated compared to 86.6% in control cells), but only a few cells weakly expressed the ductal markers CK7 or CK20. However, no transferrin positive cells were observed, suggesting the absence of cells with a hepatocyte phenotype (Figure 2 and Figure 3A).

In order to determine the number of cells expressing ductal markers, we counted the total number of cells in randomly chosen fields and calculated the percentage of those cells expressing the markers CK7, CK20, PNA and OV6 (Figure 3B). In Dex/EGF samples there was a significant increase in the percentage of cells expressing CK20 (p = 0.0043), PNA (p = 0.0098) and OV-6 (p<0.0001) compared to treatment with Dex or EGF alone. The percentage of cells expressing CK7 was also higher in Dex/EGF treated samples compared to controls (Figure 3B).

Since the combined treatment of Dex and EGF considerably enhanced the binding sites for PNA, we focused on quantifying the percentage of cells expressing high levels of PNA in Dex/EGF-treated cultures using a FACScanto cell sorter. PNA was used to sort the cells as this cell surface marker allows labelling of the cells without compromising cell integrity. Cells were labelled with a FITC-conjugated PNA antibody and sorted according to the FITC intensity. Approximately 24% of cells treated with Dex/EGF were intensely FITC positive (Figure 3C) and likely represent the ductal cells exhibiting strong PNA by immunostaining. In order to determine the expression of ductal markers in a more homogeneous population of cells, we used Magnetic activated cell sorting (MACS) to enrich for a population of ductal cells. Cells were labelled with biotinylated-PNA and sorted using the MiniMACS system. Both positive and negative cell fractions were collected and the cells returned to culture in the presence of EGF. Sorted cells were immunostained for CK7 and Sox9. PNA directed enrichment of ductal cells was confirmed in the positive cell fraction in which 25% of cells stained positive for the ductal marker CK7 (p = 0.0079) and 82% for the ductal specific transcription factor Sox9 (p = 0.0028) (Figure 3C).

To confirm the increase in ductal cells was due to the specific affect of EGF treatment, we added the EGF receptor inhibitor AG1478 to pancreatic cells from day 1 of EGF treatment. We were able to inhibit ductal cell formation as indicated by the loss of CK7 expression in inhibitor-treated samples (Figure 4C).

We have also tested the ability of primary acinar cells and additional pancreatic cell lines such as Capan1 and Panc-1 to differentiate towards hepatocyte and ductal cell phenotypes. Our preliminary data suggests that neither Capan1 nor Panc1 cells can be induced with Dex to generate hepatocytes (unpublished observations). In contrast, primary mouse acinar cells transdifferentiate to generate transferrin and C/EBPβ-positive hepatocytes [10]. However, due to contamination of the primary cultures with ductal cells we were unable to determine the ability of the acinar cells to transdifferentiate to ductal-like cells.

To determine the affect of Dex/EGF treatment on the hepatic phenotype, we analysed the protein levels of the liver markers alpha-fetoprotein, albumin, transferrin and the transcription factor C/EBPβ by Western blotting (Figure 4B). Albumin, AFP and TFN were induced in both Dex and Dex/EGF treated cells (Figure 4B), however, liver protein levels were much higher in Dex-treated compared to those treated with Dex/EGF. Furthermore, the liver enriched transcription factor C/EBPβ was detected only in the Dex treated cells (data shown for 10 day treatment only).

### Ultrastructure of Ductal-like Cells

**Figure 5:**
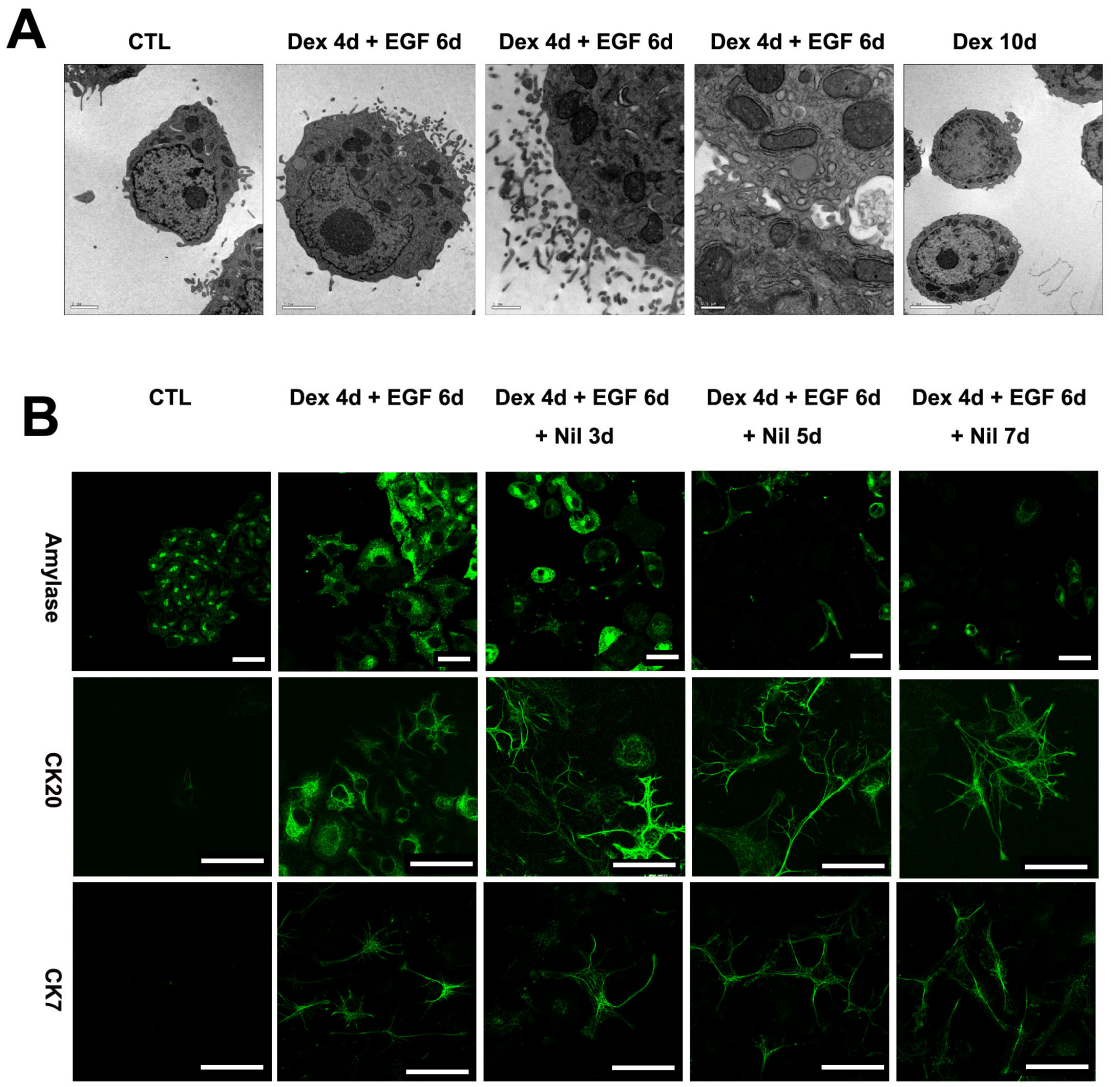
Electron microscopy and stability of the ductal phenotype. (A) Electron micrographs of control, Dex and Dex/EGF treated B13 cells. (B) Immunostaining for amylase, CK20 and CK7 following withdrawal of Dex and EGF in treated B13 cells. Control B13 cells are also shown (B). Scale bars for electron micrographs are (from left to right); 2, 2, 1 and 0.5 µm. Scale bars in second row, 20 µm and 40 µm for all others.

We examined the ultrastructural characteristics of the Dex and EGF treated cells by electron microscopy to identify the subcellular morphology of ductal type cells. The presence of prominent microvilli along the plasma membrane in isolated and cultured adult ducts has been described [25] and such structures are essential to the normal function of these cells *in vivo* [26]. Control and Dex-treated cells possessed only small sparse microvilli while we observed well-formed microvilli projecting from the surface of Dex/EGF treated cells (Figure 5A). Given that our cultures are heterogeneous (containing undifferentiated B13 cells, reprogrammed hepatocytes as well as ductal cells) and the sample size for EM analysis is very small, it was not possible to quantify cell numbers to directly compare with immunostaining data.

### Stability of the Ductal Phenotype

We were interested to know whether the ductal phenotype was dependent upon the continued presence of Dex and EGF or whether the reprogrammed ductal cells are stable and unable to revert to the parent cell of origin. To address this question, we examined the stability of the ductal cells after withdrawal of Dex and EGF. B13 cells were treated initially for 4 days with Dex and 6 days with EGF, the EGF was then withdrawn and cells fixed at 3, 5 and 7 days later and stained for amylase, CK7 and CK20. Cells positive for CK7 and CK20 were still present 7 days after discontinuing EGF treatment as judged by immunofluoresence (Figure 5B) and the amylase-expressing cells did not increase significantly. This suggests that, once induced, the ductal phenotype is stable (at least for the time points examined) in the absence of Dex and EGF.

### Exocrine cells are precursors of the ductal cells

**Figure 6:**
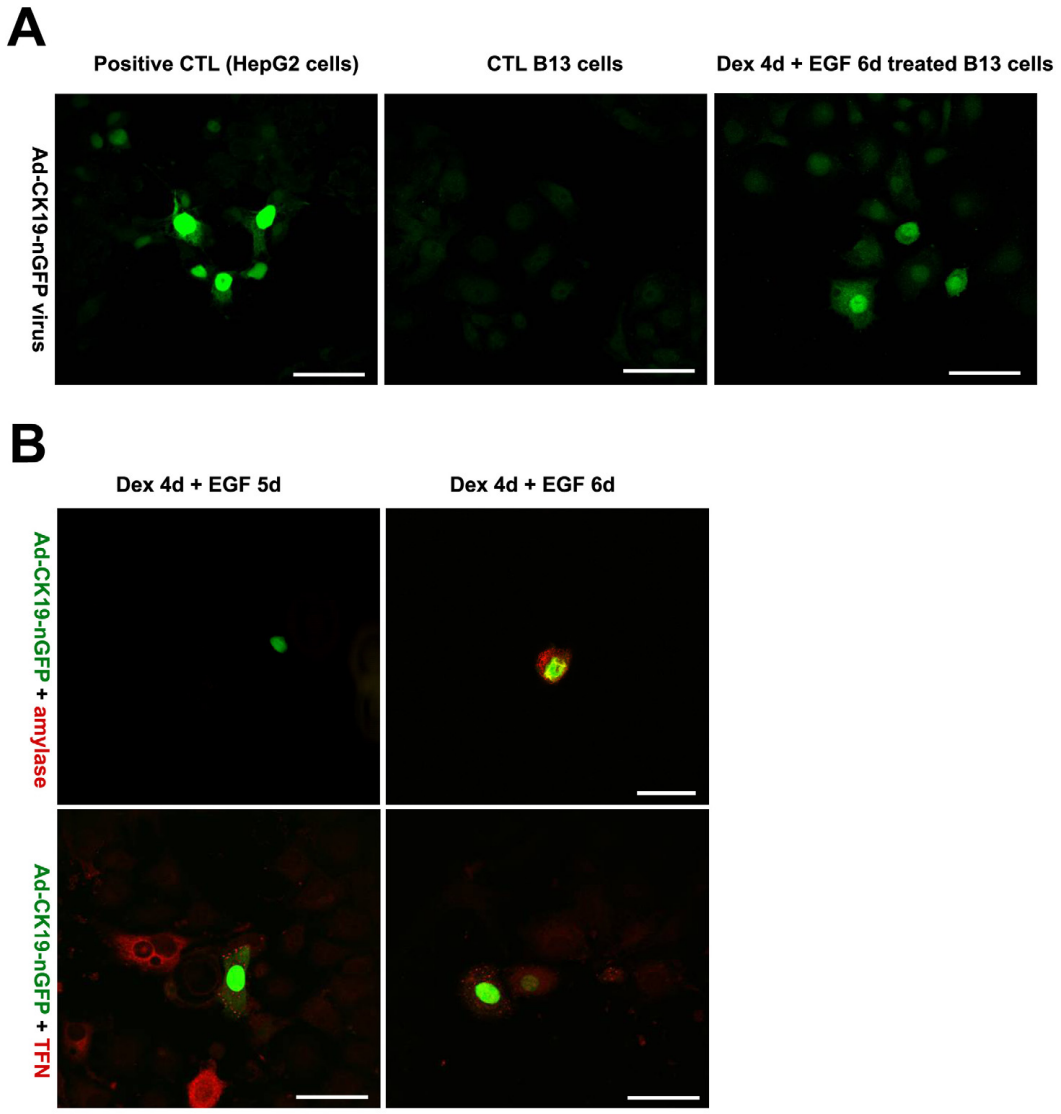
Lineage trace of ductal phenotype. (A) Infection of HepG2, control B13 and Dex/EGF treated B13 cells with Ad-CK19-nucGFP and (B) immunostaining for amylase and TFN in Dex/EGF treated B13 cells infected with Ad-CK19-nucGFP.

Since Dex treatment also induced the hepatocyte phenotype, ductal cells might arise from these cells or directly from exocrine cells. To distinguish some of these possibilities and determine the origin of these cells, we co-stained for exocrine and hepatocyte or ductal markers. A transitional expression of both markers would be expected if the ductal cells arise from the exocrine cells by direct conversion. Due to the requirement for different fixatives we were unable to stain cells for amylase (paraformaldehyde fixation) and CK7 (acetone:methanol fixation). Therefore, we used an alternative approach to determine the ductal cell lineage. Ductal cells were traced using an adenoviral reporter construct in which the CK19 promoter was used to drive GFP expression. Since CK7 and CK19 occur as a heterotypic pair and both exhibit specificity for ductal cells types (Figure1), we used the CK19 promoter to drive the GFP reporter. The fidelity of expression of the CK19 promoter construct was tested by infection of control B13 cells (negative control) and HepG2 cells. HepG2 cells were used as a positive control since the hepatoma expresses CK19 [27]. HepG2 cells were positive for GFP suggesting the promoter is active in these cells (Figure 6A) while the control B13 cells (which do not normally express CK19) were negative for GFP (Figure 6A), thus confirming there was no ‘leakiness’ of promoter expression. GFP expression was detected in Dex/EGF treated cells indicating that cells with a ductal phenotype are present and capable of activating the CK19 promoter. The adenoviral vector was introduced into B13 cells after 4 days of Dex and 2 days of EGF treatment presumably when the cells were just switching from one phenotype to another. B13 cells infected with AdCK19-nucGFP were cultured for up to 4 days, then fixed and stained for the exocrine marker amylase. We found that a subpopulation of GFP-expressing cells still expressed amylase (Figure 6B). While no GFP positive cells were found to express the hepatic marker TFN. Although we cannot exclude the possibility that EGF is causing proliferation of existing ductal cells within the culture, this data suggests that at least some of the ductal cells may arise directly from exocrine cells but not from hepatocytes. We also observed GFP positive/amylase negative cells. These cells may have lost their amylase expression at the time of analysis.

### Role of CCAAT enhancer binding protein β in formation of ductal cells

**Figure 7:**
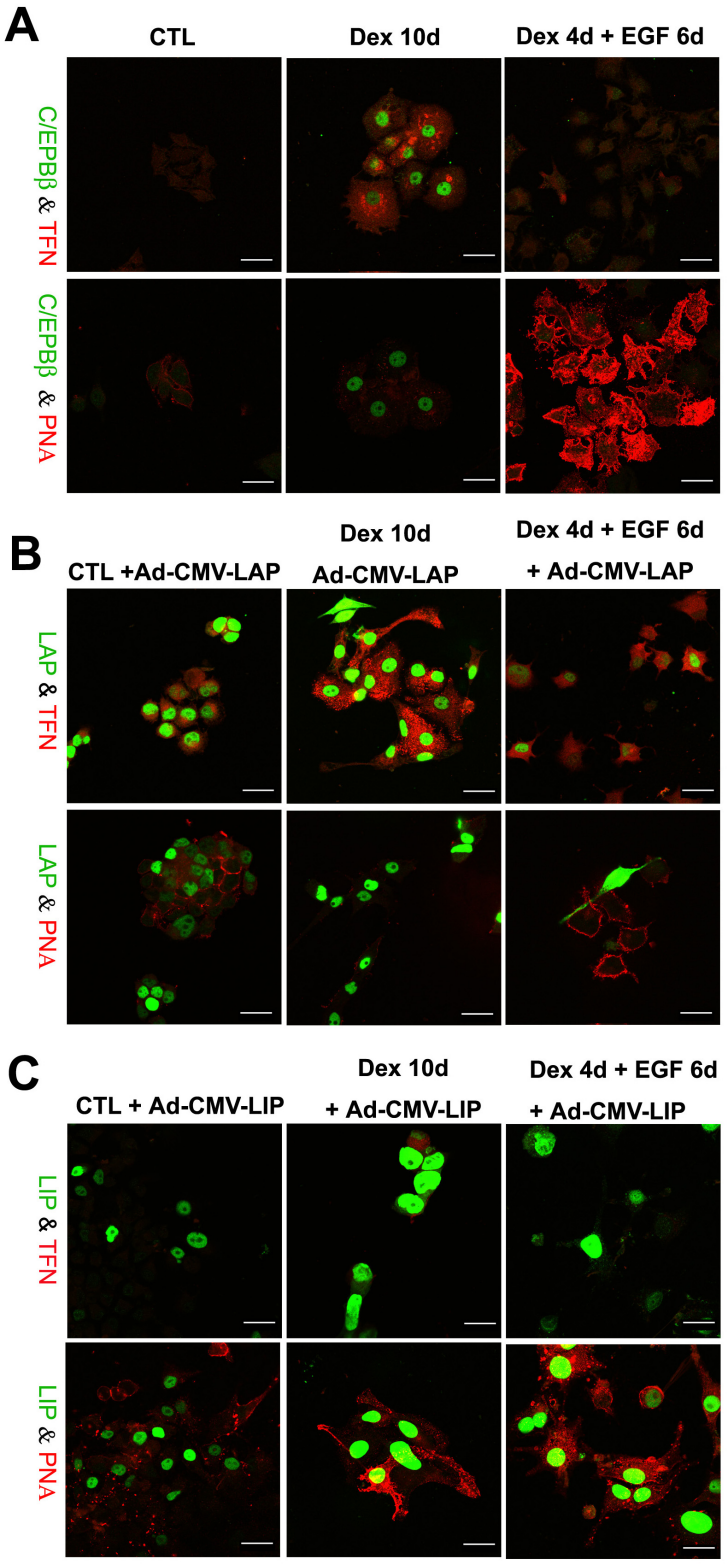
CEBPβ controls the switch in phenotype from pancreatic B13 cells to hepatocyte or ductal cells. Immunostaining for C/EBPβ/TFN and C/EBPβ/PNA in control, Dex and Dex/EGF treated cells (A) and after infection with Ad-CMV-LAP (B) and Ad-CMV-LIP (C). In A only the induced endogenous C/EBPβ is visible. Scale bars, 20µ m.

The CCAAT enhancer binding proteins (C/EBP) are basic region/leucine zipper (bZIP) transcription factors expressed during differentiation of adipose tissue and liver [28]. One member of the C/EBP family, C/EBPβ, is transcribed into one mRNA which can be translated into three distinct isoforms designated C/EBPβ, Liver Activating Protein (LAP) and Liver Inhibitor Protein (LIP) [29]. The 21 kDa LIP lacks the transactivation domain of LAP and acts as a dominant-negative form of C/EBPβ by heterodimerizing with the full length C/EBPβ. We showed previously that C/EBPβ is required for the transdifferentiation of pancreatic B13 cells to hepatocytes [6], [7], [30]. To determine whether C/EBPβ is required for the formation of ductal cells, we stained control, Dex, EGF and Dex/EGF-treated cells for C/EBPβ. C/EBPβ was absent in control (Figure. 7A) and EGF-treated cells (data not shown), but present at very low levels in Dex/EGF cultures. Robust staining for C/EBPβ was detected in Dex treated cells staining positive for the liver marker TFN but not in cells staining for PNA (Figure 7A). Western blotting for C/EBPβ confirmed that C/EBPβ was expressed in Dex treated cells at a higher level than in control cells and cells treated with Dex/EGF (Figure 4B). To test whether overexpression or inhibition of C/EBPβ can influence the direction of reprogramming (i.e. hepatocyte vs ductal), we infected control, Dex and Dex/EGF-treated cells with the adenoviral vectors containing either LAP or LIP (Ad-CMV-LAP or Ad-CMV-LIP) and determined the expression of the hepatocyte marker TFN or the ductal marker PNA. The C/EBPβ antibody used in these experiments recognises the carboxyl terminus and therefore detects all three forms of C/EBPβ. In agreement with previous observations [6], LAP induced the liver marker TFN in control B13 cells and enhanced the expression of the hepatocyte marker in Dex-treated cultures. Dex/EGF treated cells (which do not normally express TFN in many cells) were induced to do so following infection with Ad-CMV-LAP (Figure 7A). Cells expressing the transgene, did not express the ductal marker PNA suggesting that overexpression of C/EBPβ can inhibit the formation of ductal cells and induce a hepatocyte phenotype. Conversely, overexpression of LIP in Dex-treated cultures inhibits hepatocyte formation and induces the ductal phenotype (Figure 7B). This suggests that LIP might inhibit endogenous C/EBPβ activity and enhance the ductal phenotype.

## Discussion

**Figure 8:**
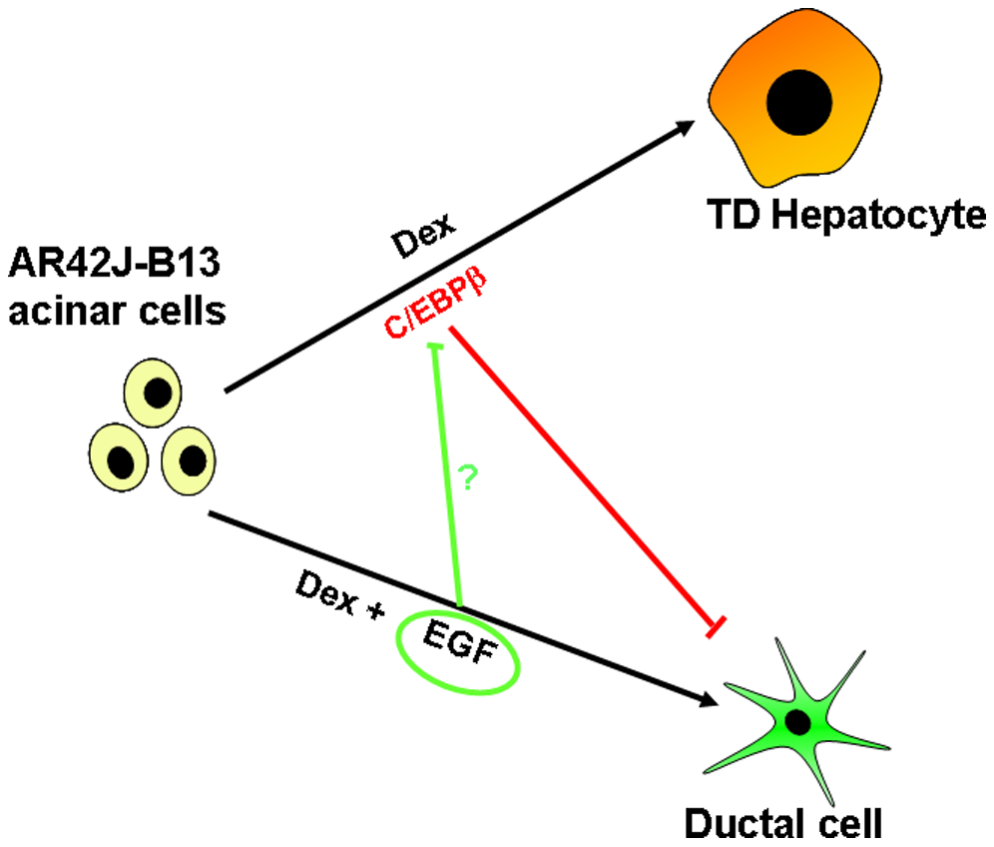
Schematic representation of the possible pathways of differentiation of B13 cells into hepatocytes and ductal cells. Diagram illustrating the potential relationship between pancreatic acinar cells, ductal cells and hepatocytes.

Acinar-ductal transdifferentiation is clinically significant as it is present in pancreatitis. The switch from acinar to ductal cell can be induced *in vivo* by pancreatic ductal ligation [21], overexpression of Pdx1 [31], or *in vitro* by the addition of external factors (DMSO) [32] or EGF [19], [24]. In the present study, we demonstrate that cells resembling a ductal phenotype are also induced following treatment of pancreatic B13 cells with Dex. The efficiency of conversion to a ductal phenotype is very low but can be enhanced by a combined treatment of Dex followed by EGF. Dex treatment has been shown previously to induce the conversion of pancreatic cells towards a hepatocyte phenotype [6], [7], [30], [33]. Remarkably, the conversion to ductal cells is stable as ductal cells maintained their phenotype for at least for 7 days after withdrawal of EGF. These observations suggest a bistable switch operates in which pancreatic B13 cells can generate either hepatocytes or ductal cells following Dex treatment. Due to the acinar nature (amylase-expression) of the B13 cells it is possible that the cells can undergo acinar-ductal metaplasia similar to that described in a number of other systems [20], [32], [34]. The evidence for a genuine acinar-ductal conversion in the B13 cell model is threefold. First, the typical duct cytokeratin CK7 is induced after treatment and are not present in control cells. Moreover, other duct and progenitor markers such as CK20, OV-6 and PNA are increased in Dex treated compared to untreated cultures. Second, morphological and ultrastructural features reminiscent of ductal cells (such as well-formed microvilli) are present. Third, we were able to identify a population of cells co-expressing amylase and CK19 (as shown by a GFP reporter construct) which indicates an intermediate population of amylase expressing cells may generate at least some of the ductal cells. At the same time there were no cells co-staining for hepatocyte markers and CK19. Therefore, we propose that ductal cells can arise from amylase-positive acinar cells following treatment with Dex and that ductal cells form a separate population of cells independent of the reprogrammed hepatocytes (Figure 8).

The question arises: what is the mechanism of the increase in ductal cells following EGF treatment? At least three possibilities exist. The first is that EGF acts directly on ductal cells formed during reprogramming of acinar cells to promote proliferation. The second is that EGF promotes cell death of non-ductal cells increasing the overall proportion of ductal cells. The third possibility is that EGF directly promotes the reprogramming of pancreatic acinar cells to ductal cells. Using the existing model it is not possible to distinguish which of these mechanisms are in operation. Further studies are required to elucidate the cellular basis underpinning the role of EGF in enhancing ductal cell numbers.

Previous studies have revealed the role of transcription factors inducing acinar-ductal conversion *in vivo* and *in vitro*. For example, ectopic expression of Pdx1 in acinar cells has been shown to reprogram cells to a ductal phenotype through activation of the Stat3 pathway in the mouse [31]. In our model, we were particularly interested in the role of the liver-enriched transcription factor C/EBPβ. Previous work by our lab [6] suggested that C/EBPβ is required for transdifferentiation of pancreatic B13 cells to hepatocytes. As expected, C/EBPβ levels were much higher in cells treated with Dex compared to Dex/EGF treated cells. This difference is probably due to the fact that fewer hepatocyte-like cells are induced following treatment with Dex/EGF.

We found that the C/EBPβ splice variants LAP and LIP (liver-activating and inhibitory proteins) play important roles in switching B13 cells towards a ductal or hepatic cell type. Adenoviral-mediated overexpression of LAP reprogrammed B13 cells towards a hepatocyte phenotype. PNA stained cells were devoid of LAP suggesting that LAP suppresses the ductal phenotype and promotes the hepatic phenotype. In contrast, cells overexpressing LIP were PNA positive suggesting that LIP promotes ductal rather than hepatic differentiation under Dex-treated conditions. LIP, which lacks the transactivation domain of LAP, may antagonize the effect of low levels of endogenous C/EBPβ present in B13 cells and promote their ductal differentiation.

We propose that in addition to hepatocytes, ductal-like cells are formed following Dex treatment of pancreatic B13 cells. Ductal-like cells expressed CK7, CK19, CK20, Cx43, PNA and OV6. We demonstrated that Dex/EGF treatment activated the CK19 promoter in amylase expressing cells suggesting an ancestor-descendent relationship between amylase-positive cells and ductal cells. Furthermore, the hepatic master switch gene C/EBPβ appears to be reduced during the induction of ductal cells and when overexpressed inhibits the ductal phenotype. This data suggests that C/EBPβ is the master gene for hepatocytes.

## Materials and Methods

### Ethics Statement: Animals

All animal experiments were performed under the UK Home Office guidelines. The handling of animals involved Schedule 1 killing by authorised personnel and thus did not require protocol review.

### Cell culture conditions

AR42J-B13 cells were a generous gift from Professor Itaru Kojima (Institute for Molecular and Cellular Regulation Gunma University, Gunma, Japan. AR42J-B13 cells were cultured in Dulbecco's Modified Eagle's Medium (DMEM) (Sigma) as described previously [6]. For immunostaining, cells were seeded at 10–15% confluency on sterile glass coverslips (22 mm×22 mm). Cells were cultured at 37°C in a humidified incubator with 5% CO2 and 95% air. To induce transdifferentiation, cells were treated with 1 µM Dex (Sigma, Poole, UK) for 10 or 14 days, 1 µM Dex for 4 days then with 20 ng/ml of EGF (R&D systems, Abingdon, UK) for 6 days, or 20 ng/ml EGF alone for 10 days. To inhibit the EGF signalling pathway the EGF receptor inhibitor AG1478 (Calbiochem) (final concentration of 25 µM) was added to cultures at the same time as EGF and cultured for 6 days.

### Immunofluorescent staining of cell cultures

B13 cells were immunostained as described previously [35], [36] with the following modifications. Cells were fixed either with 4% paraformaldehyde (PFA) (Fisher scientific, Leicestershire, UK) in PBS, pH 7.4 for 20–30 mins at room temperature or ice cold acetone:methanol (Ac:Me, 1∶1) for 5 mins depending on the antibody under investigation. In some cases, PFA-fixed cells were post-fixed with Ac:Me for 5 minutes. Prior to immunostaining, PFA fixed cells were permeabilised in 0.1% (v/v) Triton X-100 (Sigma, Poole, UK) for 30 min. Permeabilisation was not necessary for cells fixed with Ac:Me. Antigen retrieval was then performed on Ac:Me fixed cells using either 1x (v/v) EDTA or citrate buffer (Lab vision, Newmarket, UK) at 37°C. Non-specific binding sites were blocked for 30 min in 2% blocking buffer (Roche) or 10% normal goat serum (NGS). Primary antibodies and dilutions used were: rabbit-anti-amylase (1∶100, Sigma, Poole, UK), mouse-anti-cytokeratin (CK) 7 (used neat, a generous gift from Dr. Birgit Lane or Abcam 1∶40, Cambridge, UK), mouse-anti-CK20 (1∶50, DAKO, High Wycombe, UK), mouse and rabbit-anti-C/EBPβ (1∶100, Santa Cruz Biotechnology, CA, USA), mouse-anti-OV6 (1∶3000, a generous gift from Dr. Stewart Sell, Albany Medical College) and rabbit-anti-transferrin (TFN) (1∶200, DAKO, High Wycombe, UK), mouse-anti-Connexin 43 (Cx43) (1∶25, Sigma, UK). The fluorescein conjugated lectin, peanut agglutinin (PNA) (1∶100, Vector Laboratories, Peterborough, UK), was also used to stain cells. Secondary antibodies used were rabbit-anti-sheep-Texas Red, horse-anti-mouse-fluorescein isothiocyanate (FITC), goat-anti-rabbit-FITC, horse-anti-mouse-Texas Red and goat-anti-rabbit-Texas Red (all 1∶200, Vector Laboratories, Peterborough, UK). Images were collected on a Zeiss LSM 510 confocal microscope and collated into Figures in Adobe Photoshop 7.0. Cells were counted manually as percentages of positive cells per field. Five fields were counted per sample and each sample was performed in triplicate. Statistical differences between treatments was determined using the un-paired Student *t*-test in GraphPad Prism software version 4.03 (GraphPad Software, San Diego, CA, USA).

### Immunohistochemistry of adult rat tissue

Following cervical dislocation, adult rat pancreatic and liver tissue were removed, washed in PBS and fixed with either Ac:Me for 1 hour or 4% PFA over night. The tissue samples were then washed again three times in PBS and left in 70% ethanol for 1 to 3 hours before processing. Fixed tissue was paraffin embedded, sectioned, dewaxed and rehydrated. PFA fixed pancreas and liver sections were permeabilised with 0.5% (v/v) Triton X-100 for 30 minutes. Antigen retrieval was then performed using either 1x (v/v) EDTA or citrate buffer for 30 minutes at 90°C. Afterwards, the slides were allowed to cool for a further 30 minutes at room temperature. Sections were incubated with peroxidase block solution (DAKO Envision peroxidase system, DAKO) to prevent endogenous peroxidase activity. Non-specific binding was blocked by either adding 2% blocking buffer or 10% (v/v) NGS and 0.5% (v/v) bovine serum albumin (BSA) in PBS. Primary antibodies were diluted as follows: mouse-anti-CK7 (1∶50), mouse-anti-CK20 (1∶50), mouse-anti-OV6 (1∶5000) all diluted in 1% (v/v) normal goat serum and 0.5% (v/v) BSA in PBS. The signal was detected using the 3,3′ Diaminobenzidine (DAB) substrate-chromogen kit specific for mouse or rabbit (DAKO Envision peroxidase system, DAKO) and the sections were counterstained with hematoxylin before mounting.

### Western blotting

Protein samples were prepared from cells by freeze-thawing 4 times in a Tris-HCl lysis buffer containing 1∶100 dilution of protease inhibitor cocktail (Sigma, UK). 10 µg of each sample was run on a 10% Tris-HCl Criterion™ precast gel and subsequently transferred to nitrocellulose membrane using the Bio-Rad Criterion blotter system as described previously [7]. Blots were probed with the following antibodies diluted in 3% marvel in PBST (PBS with 0.1% v/v Tween-20, Sigma, UK); mouse-anti-α-tubulin (1∶2000, Sigma,UK), sheep-anti-albumin (1∶4000, Biogenesis), rabbit-anti-alpha fetoprotein (1∶1000, DAKO), rabbit-anti-TFN (1∶2000) and mouse-anti-C/EBPβ (1∶2000). Secondary antibodies used were rabbit-anti-sheep Horse radish peroxidase (HRP) (1∶2000, Abcam), goat-anti rabbit HRP (1∶2000, Vector) and horse-anti-mouse HRP (1∶2000, Vector).

### RT-PCR

Total RNA was isolated using Tri-reagent according to the manufacturer's instructions. cDNA synthesis from 2 µg RNA was carried out using the Superscript™ First strand Synthesis System (Invitrogen). PCR was performed using 1 µl of cDNA, ReddyMix™ Master mix (ABgene) and primers for β-actin (F:TCCGTAAAGACCTCTATGCC, R: AAAGCCATGCCA AAT GTC TC – 56°C) GSTπ (F:TGGAAGGAGGAGGTGGTTAC, R: TGTCCCTTCGTCCACTACTG – 54°C) and Cx43 (F:TCTTCATGCTGGTGTC R: TAACCAGCTTGTACCCAGG – 60°C). The conditions for amplification were as follows: initial denaturation at 95°C for 2 min, 25–35 cycles of 94°C for 1 min, 54–60°C for 1 min, 72°C for 1 min, and a final extension at 72°C for 10 min.

### Fluorescence Activated Cell Sorting (FACS) and Magnetic Activated Cell Sorting (MACS)

The BD FACScanto was used to determine the percentage of cells with the highest intensity of staining for PNA. Cells were labelled with FITC-conjugated PNA (1∶10) for 15 min prior to counting in the BD FACScanto. Cells are non-recoverable following analysis using this protocol, therefore using the appropriate dilution of PNA:buffer ratio determined by the BD FACScanto (1∶10) the cells were sorted using the MiniMACS separation system. Briefly, cells were labelled with biotinylated-PNA (1∶10) in MACS buffer (2 mM EDTA, 0.5% BSA in PBS) for 15 min at 4°C, washed in MACS buffer and collected by centrifugation at 112×*g* for 4 min. The cell pellet was resuspended in MACS microbeads conjugated to mouse anti-biotin and incubated for 15 min at 4°C. The cells were then washed and resuspended in MACS buffer. The MiniMACS column and separator were set up according to manufacturers' instructions and the microbead/cell mixture added to the column. The negative fraction was allowed to flow through and collected for further analysis. The column containing the PNA positive cells was washed 3 times with MACS buffer. To collect the positive fraction the column was removed from the magnetic field and 1 ml of MACS buffer added. The column plunger was used to force the cells out of the column. Both negative and positive fractions collected were returned to culture for 2–3 days in the presence of EGF as described above.

### Construction of a CK19-promoter GFP adenovirus

A replication-defective first-generation adenovirus was generated to express green fluorescent protein (GFP) under the control of a ductal-specific CK19 promoter [37], [38]. To clone the adenoviral vector, the CMV promoter from pcDNA3 CMV nucGFP plasmid (GFP under the control of a nuclear localization signal) was replaced by a 2.1 kb BamHI fragment containing the CK19 promoter elements. A CK19-nucGFP SalI, XbaI fragment of the resulting construct was then subcloned into a promoterless pShuttle vector (AdEasy kit, Stratagene) digested with XhoI and XbaI. The resulting 9.6 kb construct was recombined into a 33.5 kb pAdEasy plasmid (Stratagene) containing the majority of the adenoviral genome. Recombinants were selected according to the manufacturer's instructions. The Ad-CK19-nucGFP adenovirus was transfected and propagated in the human embryonic kidney (HEK) 293 cell line and purified as previously described [39]. The virus was titered using the Adeno-X™ Rapid Titer Kit (Clontech, CA, USA).

### Viral infection of B13 cells

B13 cells were infected with the adenovirus Ad-nucCK19-nGFP after 6 days of treatment (Dex 4 days then EGF 2 days) at a multiplicity of infection (MOI) of 200 and incubated overnight in 95% air, 5% CO2 at 37°C. The next day the media was changed and EGF treatment of the cells continued for a further 3 days. Following 4 days of Dex treatment B13 cells were also infected overnight with either the liver activating protein (LAP) or the liver inhibitory protein (LIP) both at an MOI of 50. Dex treatment was either continued or switched to EGF for an additional 6 days. The cells were then PFA fixed and permeabilised with 0.1% (v/v) Triton X-100 for 30 minutes before staining.

### Electron Microscopy

Control and treated B13 cells were processed for electron microscopy. Samples were fixed in 2.5% (v/v) glutaraldehyde (Agar Scientific, Essex, UK) in culture medium without serum for 2 hours. Postfixation took place in a solution of 1% (w/v) potassium ferrocyanide in 1% (v/v) aqueous osmium tetroxide (both from Agar Scientific, Essex, UK). Cell pellets were encapsulated in agarose (Sigma, Poole, UK) and stained in 1% (w/v) aqueous uranyl acetate (Agar Scientific, Essex, UK). After dehydration in acetone (Fisher Scientific, Leicestershire, UK) samples were embedded in epoxy resin (TAAB Laboratories Equipment, Berks, UK). Sections were viewed in a JEOL 1200Ex transmission electron microscope (JEOL, Tokyo, Japan) operating at 80 kv.
