# Correction: Dexamethasone Treatment Induces the Reprogramming of Pancreatic Acinar Cells to Hepatocytes and Ductal Cells

**DOI:** 10.1371/journal.pone.0219419

**Published:** 2019-07-02

**Authors:** Amani Al-Adsani, Zoë D. Burke, Daniel Eberhard, Katherine L. Lawrence, Chia-Ning Shen, Anil K. Rustgi, Hiroshi Sakaue, J. Mark Farrant, David Tosh

After publication of this article [1], concerns were raised about irregularities in Fig 4B and vertical lines regarding C/EBPβ.

The authors would like to make the following clarification regarding the western blotting data for C/EBPβ in Fig 4B.

The scanned image of the CEBPβ western blot was used and this data is presented with westerns for a set of liver markers (Albumin, Transferrin and alpha fetoprotein) and alpha tubulin. However, the samples for these western blots (Albumin, Transferrin, alpha fetoprotein and alpha-tubulin) were loaded in a different order to the samples in the CEBPβ western (omitting the 14day data). Therefore, the scanned image for the CEBPβ western was cropped and rearranged so that the order of the samples corresponded across all western blots included in Fig 4B. All images were adjusted to have a similar size in Photoshop (so that the lanes lined up and all panels were of a similar width and height) and then compiled into a single annotated figure. Please view the updated Fig 4 and updated legend here.

**Fig 4:**
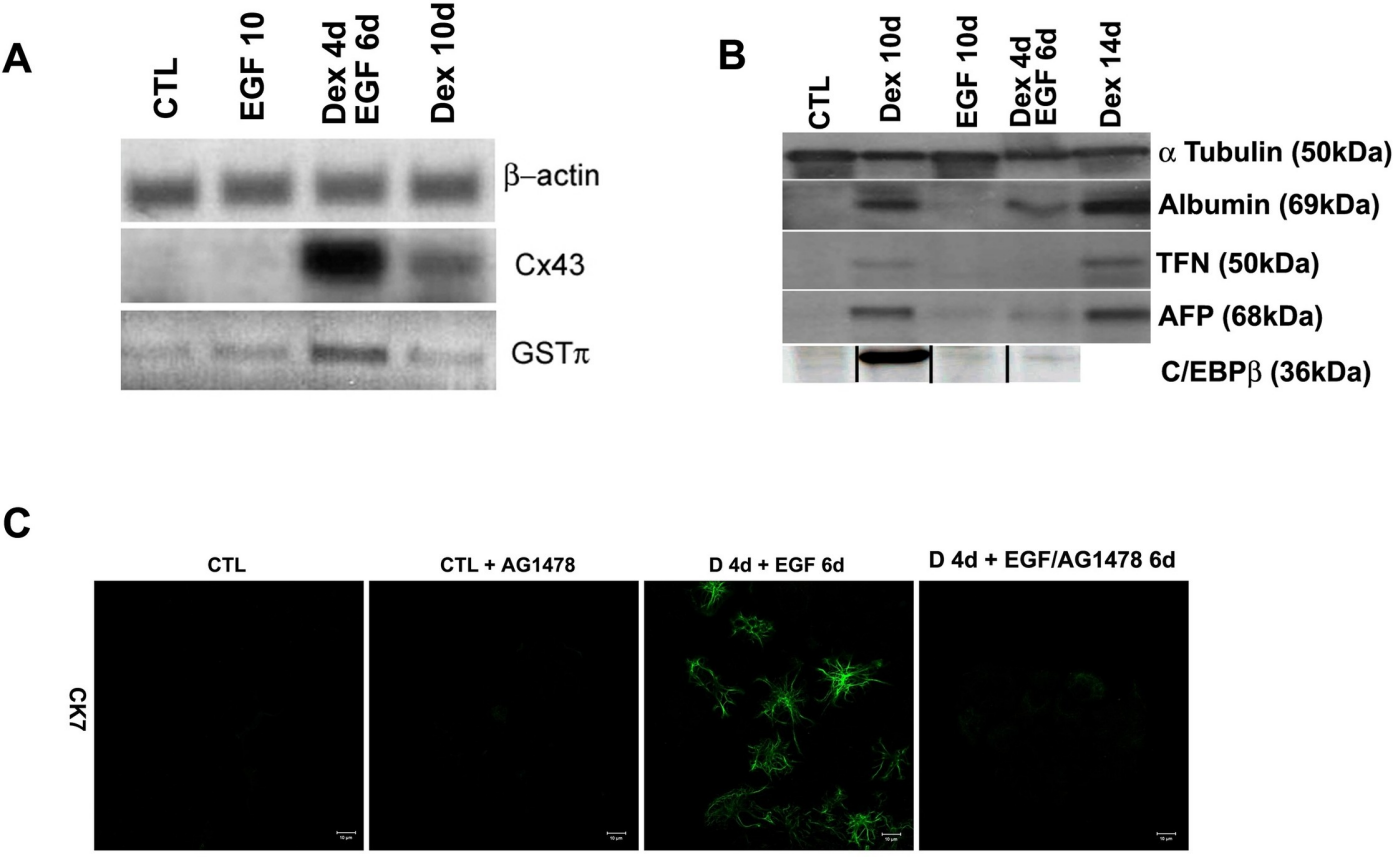
Expression of ductal markers and inhibition of the ductal phenotype. (A) RT-PCR for Cx43 and GSTπ. β-actin is also shown as a loading control. (B) Western blotting analysis for Albumin, TFN, AFP and the liver enriched transcription factor C/EBPβ in control, EGF, Dex/EGF and Dex treated cells. α-tubulin is also shown as a loading control. The original blot used to generate data for C/EBPβ was cropped and re-arranged to correspond to the loading order used in the blots for Albumin, TFN and AFP. Splicing is denoted by vertical black lines. The raw blot for C/EBPβ is provided in supporting information. (C) Immunostaining for CK7 in control and Dex/EGF treated cells in the presence and absence and absence of the EGF receptor inhibitor AG1478. The inhibitor was added at a final concentration of 25 μM.

The uncropped and unaltered images of the autoradiographs underlying all western blots included in the article are provided below as a Supporting Information file (S1 File). Additional raw data underlying Fig 4B are available at https://doi.org/10.6084/m9.figshare.7624001.v1. An additional example of CEBPβ western blotting from a repeat of this experiment (with samples loaded in the correct order) is also provided at the URL above.

## Supporting information

S1 File Raw data. (ZIP)
