# Supplementary figures and images for: Correction: Dexamethasone Treatment Induces the Reprogramming of Pancreatic Acinar Cells to Hepatocytes and Ductal Cells
Source: PLoS One. 2019 Jul 2;14(7):e0219419. doi: 10.1371/journal.pone.0219419 (PMC6605663; doi:10.1371/journal.pone.0219419)

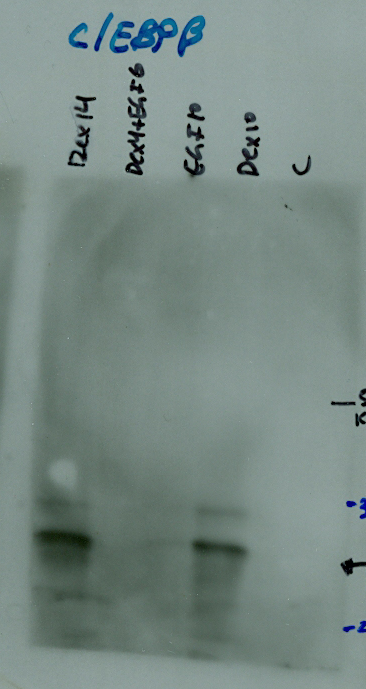

Supplement: S1 File — (ZIP) [file pone.0219419.s001.zip › S1 File/Additional CEBPb blot loaded in order.tif]

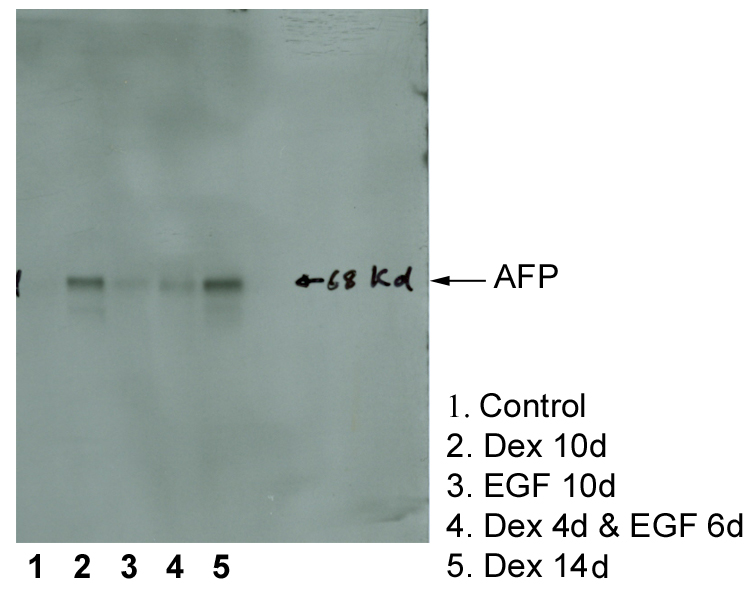

Supplement: S1 File — (ZIP) [file pone.0219419.s001.zip › S1 File/AFP Western 1.jpg]

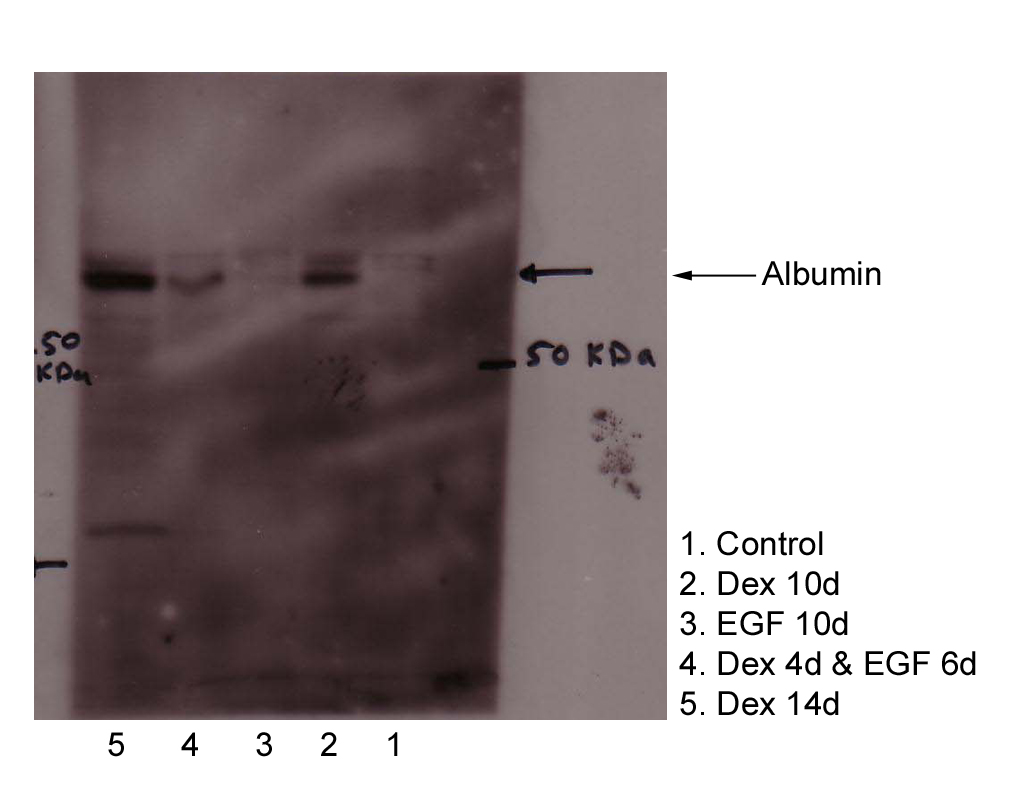

Supplement: S1 File — (ZIP) [file pone.0219419.s001.zip › S1 File/Albumin Western 1.jpg]

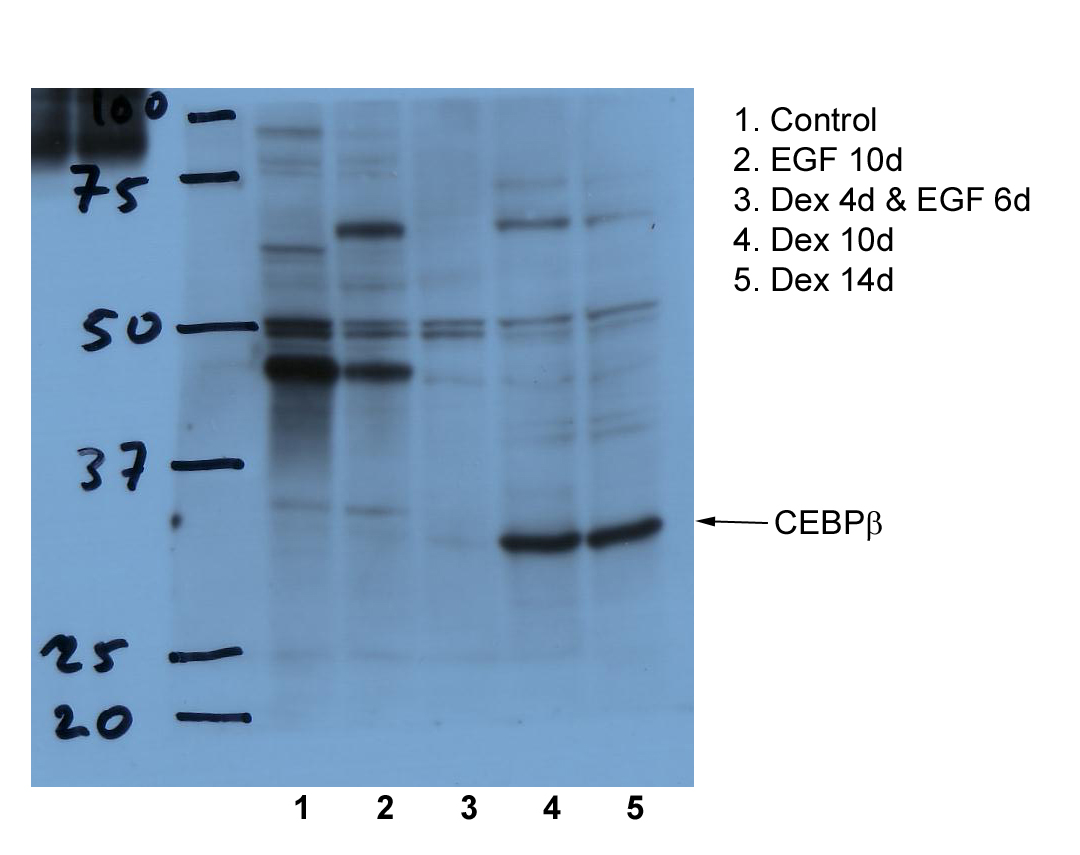

Supplement: S1 File — (ZIP) [file pone.0219419.s001.zip › S1 File/CEBPb Western 1.jpg]

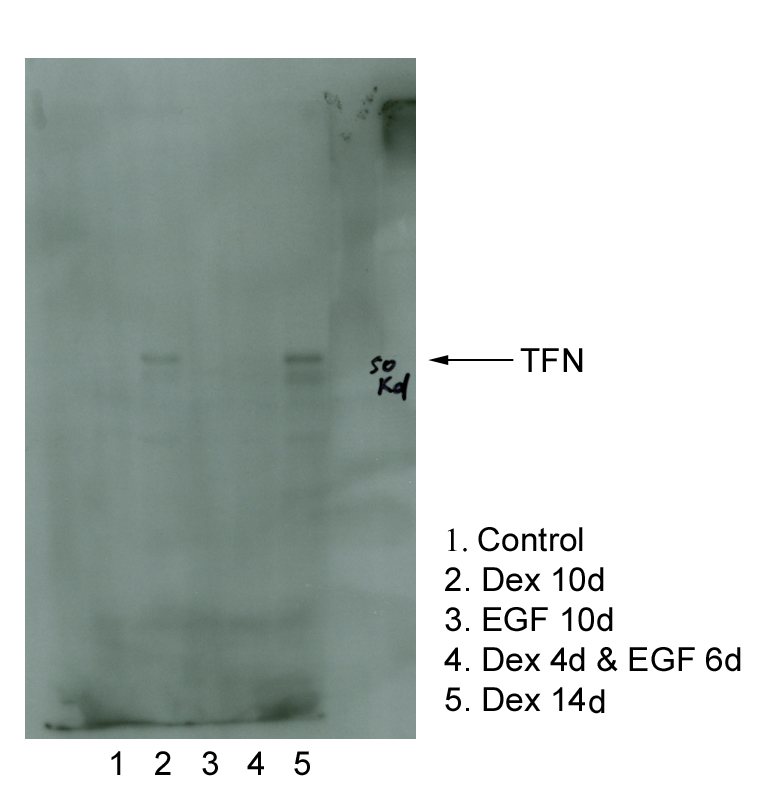

Supplement: S1 File — (ZIP) [file pone.0219419.s001.zip › S1 File/TFN Western 1.jpg]

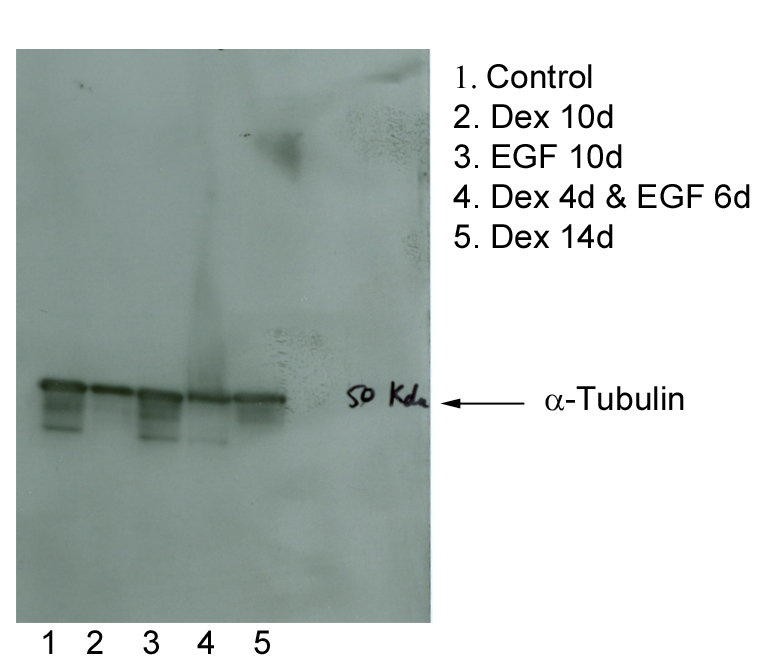

Supplement: S1 File — (ZIP) [file pone.0219419.s001.zip › S1 File/Tubulin Western 1.jpg]
